# The pro-inflammatory cytokines IL-1β and IL-6 promote upregulation of the ST6GAL1 sialyltransferase in pancreatic cancer cells

**DOI:** 10.1016/j.jbc.2024.107752

**Published:** 2024-09-12

**Authors:** Austin D. Silva, Jihye Hwang, Michael P. Marciel, Susan L. Bellis

**Affiliations:** Department of Cell, Developmental and Integrative Biology, University of Alabama at Birmingham, Birmingham, Alabama, USA

**Keywords:** glycosylation, ST6GAL1, cytokine, gene regulation, pancreatic cancer

## Abstract

The ST6GAL1 sialyltransferase is overexpressed in multiple cancers, including pancreatic ductal adenocarcinoma (PDAC). ST6GAL1 adds an α2-6-linked sialic acid to *N*-glycosylated membrane receptors, which consequently modulates receptor structure and function. While many studies have investigated the effects of ST6GAL1 on cell phenotype, there is a dearth of knowledge regarding mechanisms that regulate ST6GAL1 expression. In the current study, we evaluated the regulation of ST6GAL1 by two pro-inflammatory cytokines, IL-1β and IL-6, which are abundant within the PDAC tumor microenvironment. Cytokine activity was monitored using the Suit-2 PDAC cell line and two Suit-2-derived metastatic subclones, S2-013 and S2-LM7AA. For all three cell models, treatment with IL-1β or IL-6 increased the expression of ST6GAL1 protein and mRNA. Specifically, IL-1β and IL-6 induced expression of the *ST6GAL1* YZ mRNA isoform, which is driven by the P3 promoter. The *ST6GAL1* H and X isoforms were not detected. Promoter reporter assays confirmed that IL-1β and IL-6 activated transcription from the P3 promoter. We then examined downstream signaling mechanisms. IL-1β is known to signal through the NFκB transcription factor, whereas IL-6 signals through the STAT3 transcription factor. CUT&RUN experiments revealed that IL-1β promoted the binding of NFκB to the *ST6GAL1* P3 promoter, and IL-6 induced the binding of STAT3 to the P3 promoter. Finally, we determined that inhibitors of NFκB and STAT3 blocked the upregulation of ST6GAL1 stimulated by IL-1β and IL-6, respectively. Together, these results highlight a novel molecular pathway by which cytokines within the tumor microenvironment stimulate the upregulation of ST6GAL1 in PDAC cells.

Inflammation plays a pivotal role in the initiation and progression of pancreatic ductal adenocarcinoma (PDAC), the most common and deadly form of pancreatic cancer (1). Indeed, chronic pancreatitis is a major risk factor for PDAC development (2). During chronic pancreatitis, cytokines present within the inflammatory milieu act on pancreatic epithelial cells to impart phenotypic changes that sensitize cells to neoplastic transformation (3). Furthermore, independent of the effects of pancreatitis, tumor-elicited inflammation promotes more aggressive cancer cell behaviors that drive the transition to metastatic disease (4). Understanding the mechanisms by which inflammation fuels carcinogenesis and metastatic progression is crucial for developing more effective treatments for PDAC. However, despite intensive interest in the link between inflammation and PDAC, the role of cancer cell glycosylation remains an underinvestigated area of research.

Abnormal surface glycosylation is a hallmark feature of a neoplastic cell. In particular, changes in the composition and abundance of sialylated surface glycans are prevalent (5, 6). As one example, the sialyl Lewis a tetrasaccharide (sLe^a^, CA19–9) is elevated in pancreatitis and PDAC and is utilized as a biomarker for PDAC response to treatment and recurrence (7). Engle *et al.* developed an elegant mouse model with sLe^a^ overexpression and showed that sLe^a^ promoted the rapid development of pancreatitis and cooperated with oncogenic ras to drive aggressive pancreatic cancer (8). Another notable sialoglycan alteration in PDAC is an increase in α2-6-linked sialic acids on the *N*-glycans of glycoproteins. This modification is elaborated by the ST6GAL1 sialyltransferase (9, 10). In the normal pancreas, expression of the ST6GAL1 protein is undetectable in pancreatic acinar cells (11), a prime cell of origin for PDAC (12). In contrast, robust ST6GAL1 expression is observed in PDAC cells, and ST6GAL1 levels progressively increase as the tumor evolves from early to late stage (11). PDAC patients with high ST6GAL1 expression have a worse prognosis (13). Recently, our group discovered that ST6GAL1 is also upregulated during pancreatitis and functions within this context to promote acinar to ductal metaplasia (ADM) (11). In the process of ADM, acinar cells de-differentiate into ductal-like, progenitor cells that re-enter the cell cycle in order to effectuate tissue repair and regeneration (3, 14). However, cells undergoing ADM are particularly vulnerable to oncogene-induced transformation (3, 14). Hence, ADM is considered one of the earliest events involved in tumor initiation.

Consistent with its role in fostering ADM, ST6GAL1 promotes progenitor-like characteristics in other stemness-associated processes, including epithelial to mesenchymal transition (15, 16), acquisition of cancer stem cell properties (17, 18), and the reprogramming of somatic cells into induced pluripotent stem cells (19). One of the important features conferred by ST6GAL1 is resistance to a variety of cytotoxic stimuli (10, 20). This is accomplished through the α2-6 sialylation of membrane receptors that play crucial roles in cell survival. For instance, the α2-6 sialylation of the Fas and tumor necrosis factor receptor 1 (TNFR1) death receptors inhibits apoptosis by hindering ligand-induced receptor internalization (21, 22). The ligands for Fas and TNFR1, FasL and TNFα, respectively, are expressed at high levels within inflamed tissues (23). In addition to modulating death receptor signaling, ST6GAL1 enhances cell survival through the α2-6 sialylation of epidermal growth factor receptor (EGFR). ST6GAL1-mediated sialylation activates EGFR by promoting EGFR dimerization and receptor recycling (24), leading, in turn, to prolonged activation of survival-associated signaling cascades (24, 25). These combined effects of ST6GAL1 are expected to facilitate the survival of epithelial cells within the pancreatitis and PDAC microenvironments.

Although many studies have focused on ST6GAL1’s functional role in regulating epithelial cell phenotype, limited attention has been paid to the mechanisms governing the dynamic changes in ST6GAL1 expression that occur during pathogenesis. ST6GAL1 has a complex mechanism of gene regulation, which enables cell type–specific, tissue-specific, and disease-associated expression (20, 26). There are multiple *ST6GAL1* mRNA isoforms, with most of these transcribed from three main, spatially distinct promoters (27, 28). Each isoform has its own 5′ untranslated region (UTR), whereas the protein-coding region and 3′UTR are conserved. The P3 promoter drives transcription of the *ST6GAL1* YZ isoform, which is ubiquitously expressed (29). The P2 promoter directs transcription of the X isoform, which is restricted to B-lymphocytes (30). The P1 promoter directs transcription of the H isoform, which is selectively expressed in the liver (31, 32), as well as in some types of cancer cells (33, 34). Finally, the P4 promoter directs expression of an isoform that is only expressed in the mammary gland during lactation (35).

In light of the known upregulation of ST6GAL1 during pancreatitis and PDAC, we postulated that ST6GAL1 expression may be regulated by cytokines present within the inflamed pancreas. Accordingly, we interrogated the capacity of the pro-inflammatory cytokines IL-1β and IL-6 to induce ST6GAL1 expression. IL-1β and IL-6 are highly expressed within the tumor microenvironment (TME) and play well-established roles in PDAC development and progression (36, 37). To study cytokine-mediated regulation of ST6GAL1 expression, we used the Suit-2 PDAC cell line, along with two Suit-2-derived metastatic subclones, S2-LM7AA and S2-013. Using a combination of methods including isoform-specific RT-PCR, promoter reporter assays, and CUT&RUN experiments, we determined that expression of the YZ mRNA isoform of *ST6GAL1* is induced by the IL-1β/NFκB and IL-6/STAT3 signaling networks. These results provide new insights into the molecular mechanisms underlying the upregulation of ST6GAL1 during inflammatory processes that contribute to PDAC pathogenesis.

## Results

### Levels of IL-1β, IL-6, and ST6GAL1 are increased in tissues from patients with PDAC or chronic pancreatitis

**Figure 1:**
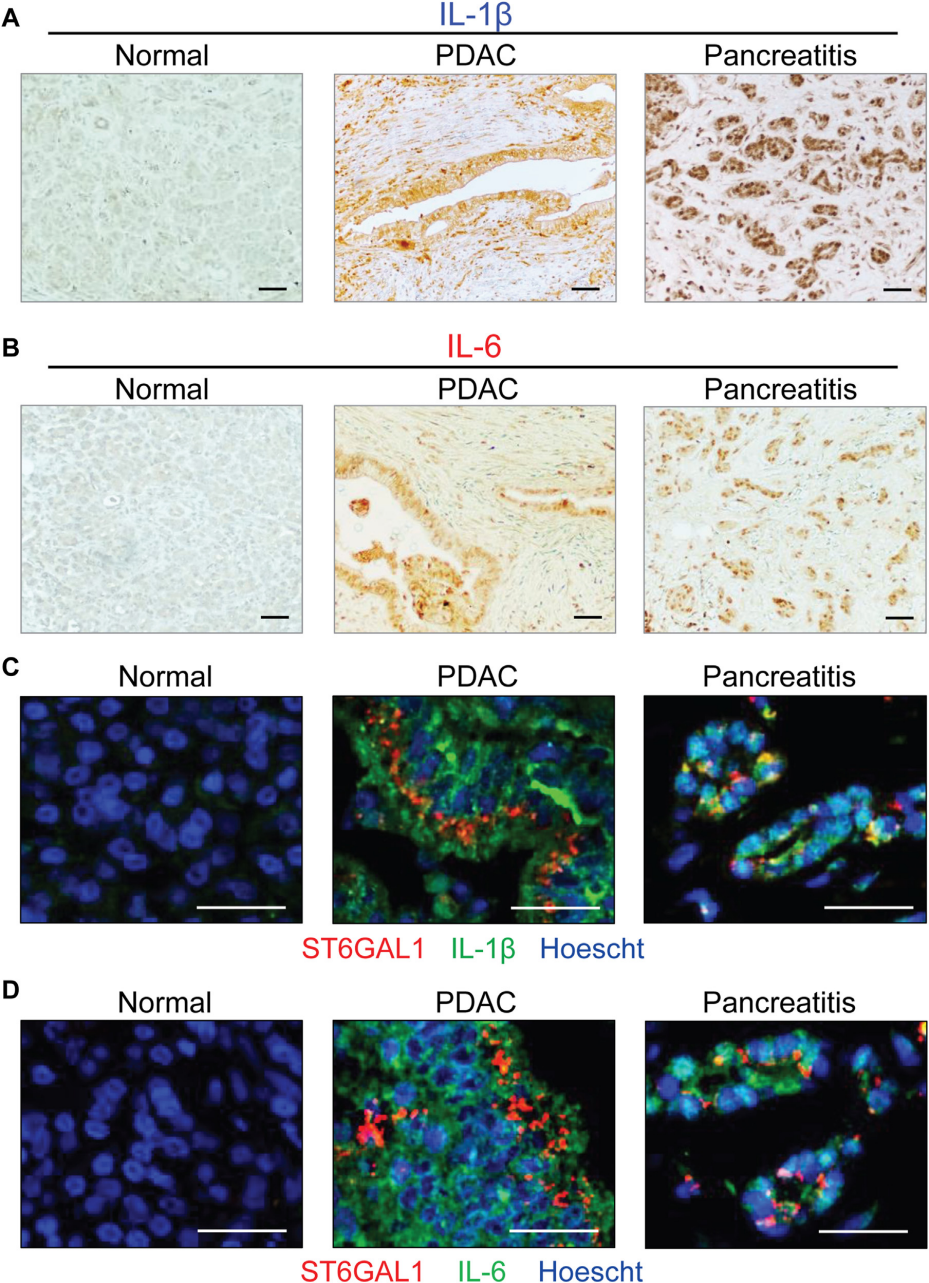
**IL-1β and IL-6 are upregulated in PDAC and pancreatitis and colocalize with ST6GAL1.***A* and *B*, IHC staining shows increased levels of IL-1β (*A*) and IL-6 (*B*) in pancreatic tissues from patients with PDAC or chronic pancreatitis when compared to adjacent normal pancreas. Scale bar = 100 μM. *C* and *D*, immunofluorescent staining of pancreatic tissues to assess expression of cytokines (*green*) and ST6GAL1 (*red*). Nuclei were stained with Hoescht (*blue*). As shown, levels of IL-1β, IL-6, and ST6GAL1 were negligible in normal pancreatic epithelium but strongly upregulated in PDAC and pancreatitis. ST6GAL1 was co-expressed with IL-1β (*C*) and IL-6 (*D*) in the epithelial compartment. Scale bar = 50 μM. PDAC, pancreatic ductal adenocarcinoma.

The expression of IL-1β and IL-6 was evaluated by immunohistochemistry on pancreata from patients with PDAC or chronic pancreatitis, or on normal human pancreas. Compared with the normal pancreas, elevated levels of IL-1β and IL-6 were observed in the PDAC and pancreatitis specimens (Fig. 1, *A* and *B*). These cytokines were expressed in both the epithelial and stromal compartments, as reported by others (38, 39). Immunofluorescence staining for cytokines and ST6GAL1 revealed that ST6GAL1 was co-expressed with IL-1β and IL-6 in the epithelial cells of PDAC and pancreatitis tissues (Fig. 1, *C* and *D*). In contrast, levels of IL-1β, IL-6, and ST6GAL1 were negligible in normal pancreatic epithelium.

### IL-1β and IL-6 induce increased expression of ST6GAL1 and α2-6 sialylation

**Figure 2:**
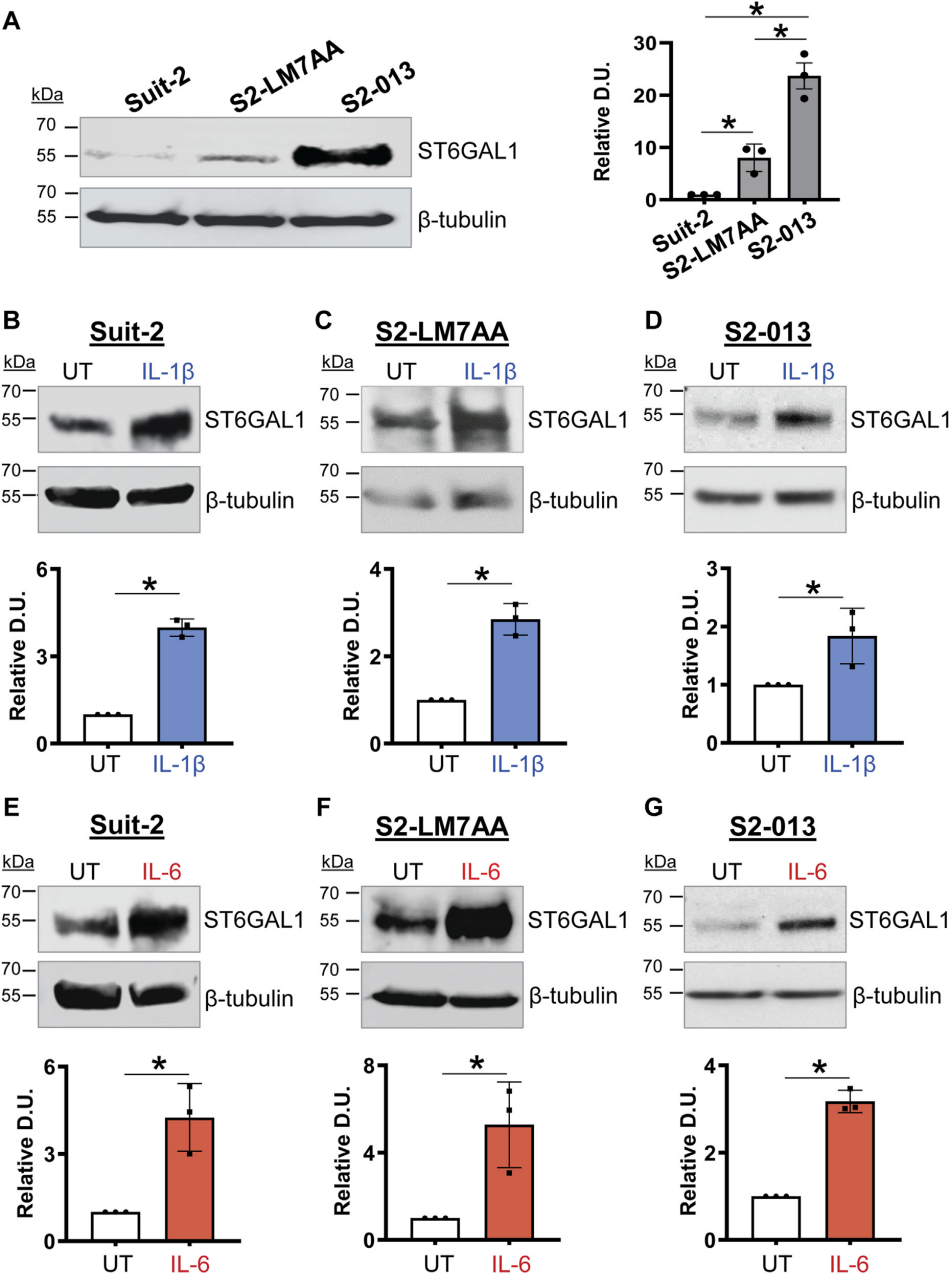
**IL-1β and IL-6 induce an upregulation in ST6GAL1 protein in PDAC cells.***A*, immunoblotting shows elevated expression of ST6GAL1 in the S2-LM7AA and S2-013 PDAC cell lines as compared with Suit-2 PDAC cells. The S2-LM7AA and S2-013 lines are metastatic subclones derived from the Suit-2 line. Densitometric units (D.U.) were normalized to β-tubulin and depicted as relative to Suit2 cells. Graphs depict mean ± S.D. (n = 3 biological replicates). Data were analyzed using a one-way ANOVA followed by Fisher’s test. ∗*p* < 0.05. *B–D*, cells were treated for 24 h with 10 ng/ml IL-1β. Lysates from cytokine-treated or untreated (UT) cells were immunoblotted for ST6GAL1. IL-1β stimulated an increase in ST6GAL1 expression in Suit-2 (*B*), S2-LM7AA (*C*), and S2-013 (*D*) cells. D.U. were normalized to β-tubulin and depicted as relative to UT cells. Graphs depict mean ± S.D. (n = 3 biological replicates). Data were analyzed using a two-tailed Student’s *t* test. ∗*p* < 0.05. *E–G*, cells were treated for 48 h with 25 ng/ml IL-6. Lysates from cytokine-treated or UT cells were immunoblotted for ST6GAL1. IL-6 stimulated an increase in ST6GAL1 expression in Suit-2 (*E*), S2-LM7AA (*F*), and S2-013 (*G*) cells. D.U. were normalized to β-tubulin and depicted as relative to UT cells. Graphs depict mean ± S.D. (n = 3 biological replicates). Data were analyzed using a two-tailed Student’s *t* test. ∗*p* < 0.05. PDAC, pancreatic ductal adenocarcinoma.

**Figure 3:**
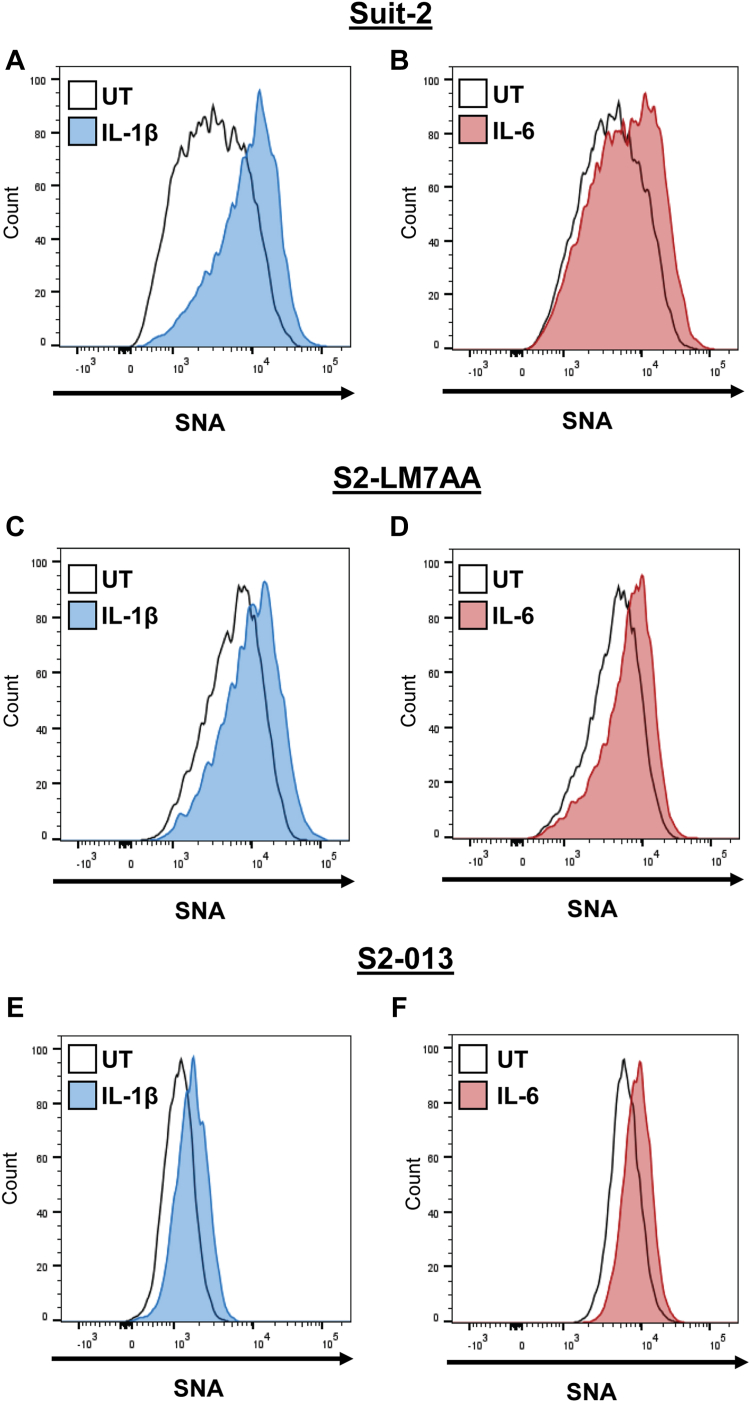
**IL-1β and IL-6 promote an increase in surface α2-6 sialylation.***A–F*, cells were treated for 24 h with 10 ng/ml IL-1β (*A*, *C*, *E*) or 48 h with 25 ng/ml IL-6 (*B*, *D*, *F*). Levels of surface α2-6 sialylation were assessed by staining with SNA followed by flow cytometry. IL-1β and IL-6 treatment resulted in increased α2-6 sialylation relative to untreated (UT) cells in the Suit-2 (*A* and *B*), S2-LM7AA (*C* and *D*), and S2-013 (*E* and *F*) lines. SNA, *Sambucus nigra agglutinin*.

To investigate whether IL-1β and IL-6 promoted the expression of ST6GAL1, we used the Suit-2 PDAC cell line, along with two metastatic subclones derived from Suit-2 cells. Among human PDAC cell lines, Suit-2 cells have unusually low levels of ST6GAL1 and limited metastatic potential (16). Other groups have used iterative *in vivo* selection methods to isolate Suit-2-derived subclones with enhanced metastatic capability (40, 41, 42). Two such subclones, S2-LM7AA and S2-013, displayed increased levels of ST6GAL1 relative to the parental Suit-2 line (Fig. 2*A*), as previously reported (16, 43). The three PDAC cell lines were treated with IL-1β (Fig. 2, *B–D*) or IL-6 (Fig. 2, *E–G*) and immunoblotted for ST6GAL1. In all of the lines, ST6GAL1 expression was strongly increased upon treatment with IL-1β or IL-6. We next evaluated whether elevated levels of ST6GAL1 were correlated with enhanced surface α2-6 sialylation. Cells were treated with or without IL-1β or IL-6 and stained with *Sambucus nigra agglutinin* (SNA), a lectin that binds specifically to α2-6 sialic acids. Flow cytometric analyses confirmed that IL-1β and IL-6 induced an increase in α2-6 sialylation (Fig. 3, *A–F*).

### IL-1β and IL-6 stimulate expression of the YZ isoform of ST6GAL1

**Figure 4:**
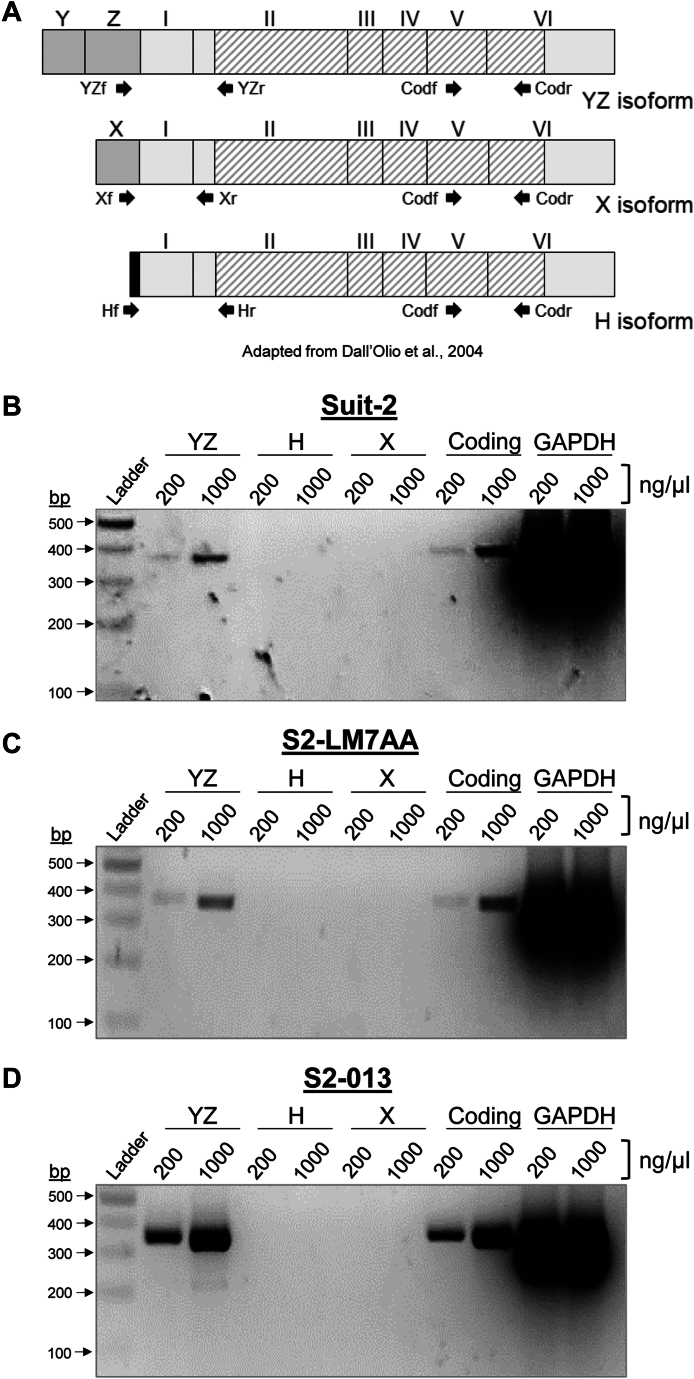
**The *ST6GAL1* YZ transcript is the most abundant mRNA isoform in PDAC cells.***A*, schematic diagram of the three main mRNA isoforms of *ST6GAL1*, and the position of the primers used for RT-PCR. The YZ isoform includes exons Y and Z, and the X isoform includes exon X. The H isoform has a unique sequence at the 5′ end of exon I (*black box*). The coding region of *ST6GAL1* spans from part of exon II to part of exon VI (*hatched region*). Primers used for RT-PCR: YZf = YZ forward; YZr = YZ reverse; Xf = X forward; Xr = X reverse; Hf = H forward; Hr = H reverse; Codf = coding forward; Codr = coding reverse. *B–D*, RT-PCR was conducted for the various isoforms, and the PCR products loaded onto gels at either 200 or 1000 ng/μl. Gels were stained with GelRed Nucleic Acid Stain to visualize the bands. For all three cell lines, Suit-2 (*B*), S2-LM7AA (*C*), and S2-013 (*D*), the YZ isoform was the only isoform detected. Expected size of the PCR products: YZ isoform = 363 bp; H isoform = 285 bp, X isoform = 334 bp, coding region = 372 bp; GAPDH = 371 bp. PDAC, pancreatic ductal adenocarcinoma.

As a first step toward delineating the mechanisms underlying ST6GAL1 upregulation, we evaluated the basal expression of the three main mRNA isoforms of *ST6GAL1*, YZ, X, and H (schematic diagram in Fig. 4*A*). All of these isoforms include exons I-VI; however, they have divergent exons upstream of exon I. The YZ isoform includes exons Y and Z, whereas the X isoform includes exon X. The H isoform differs in humans and mice. In humans, the H isoform has a unique sequence at the 5′ end of exon I, whereas mice have a distinct H exon upstream of exon I (31). The conserved coding region of *ST6GAL1* spans from part of exon II to part of exon VI. To monitor expression of the various *ST6GAL1* mRNA isoforms, primers were designed for the unique 5′UTRs, along with primers for the conserved coding region (Fig. 4*A*). Using these primers, RT-PCR was conducted on Suit-2, S2-LM7AA, and S2-013 cells. The RT-PCR reactions were resolved by electrophoresis and imaged to confirm that the amplified products were the predicted size. For all three cell lines, the YZ isoform was the only isoform detected under basal conditions (Fig. 4, *B–D*).

**Figure 5:**
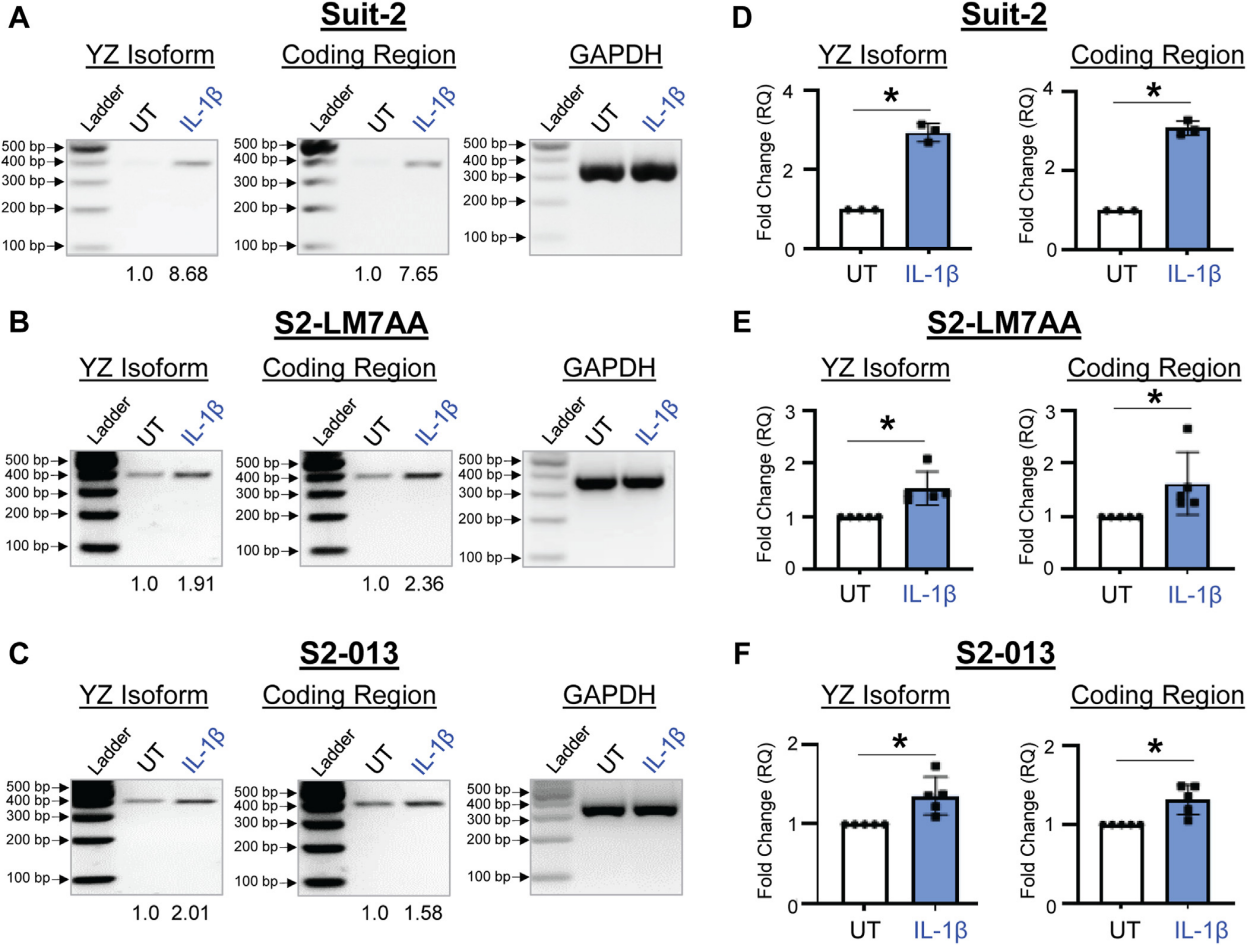
**IL-1β induces an upregulation in the YZ isoform of *ST6GAL1*.***A–C*, RNA was isolated from cells treated for 24 h with 10 ng/ml IL-1β or left untreated (UT). The YZ isoform and coding region of *ST6GAL1* were amplified by RT-PCR, and the PCR products resolved on agarose gels. Bands were quantified by densitometry. Densitometric units for the YZ and coding regions were normalized to GAPDH, and then values were reported as relative to the UT control cells. As shown, IL-1β treatment increased the expression of the YZ isoform and coding region of *ST6GAL1* in Suit-2 (*A*), S2-LM7AA (*B*), and S2-013 (*C*) cells. Predicted size of PCR products: YZ isoform = 363 bp; coding region = 372 bp; GAPDH = 371 bp. *D–F*, the YZ isoform and coding region were amplified by RT-qPCR using SYBR green. IL-1β treatment significantly increased the expression of the YZ isoform and coding regions in Suit-2 (*D*, n = 3 biological replicates), S2-LM7AA (*E*, n = 5 biological replicates), and S2-013 (*F*, n = 5 biological replicates) cells. Graphs depict mean ± S.D. Data were analyzed using a two-tailed Student’s *t* test. ∗*p* < 0.05.

**Figure 6:**
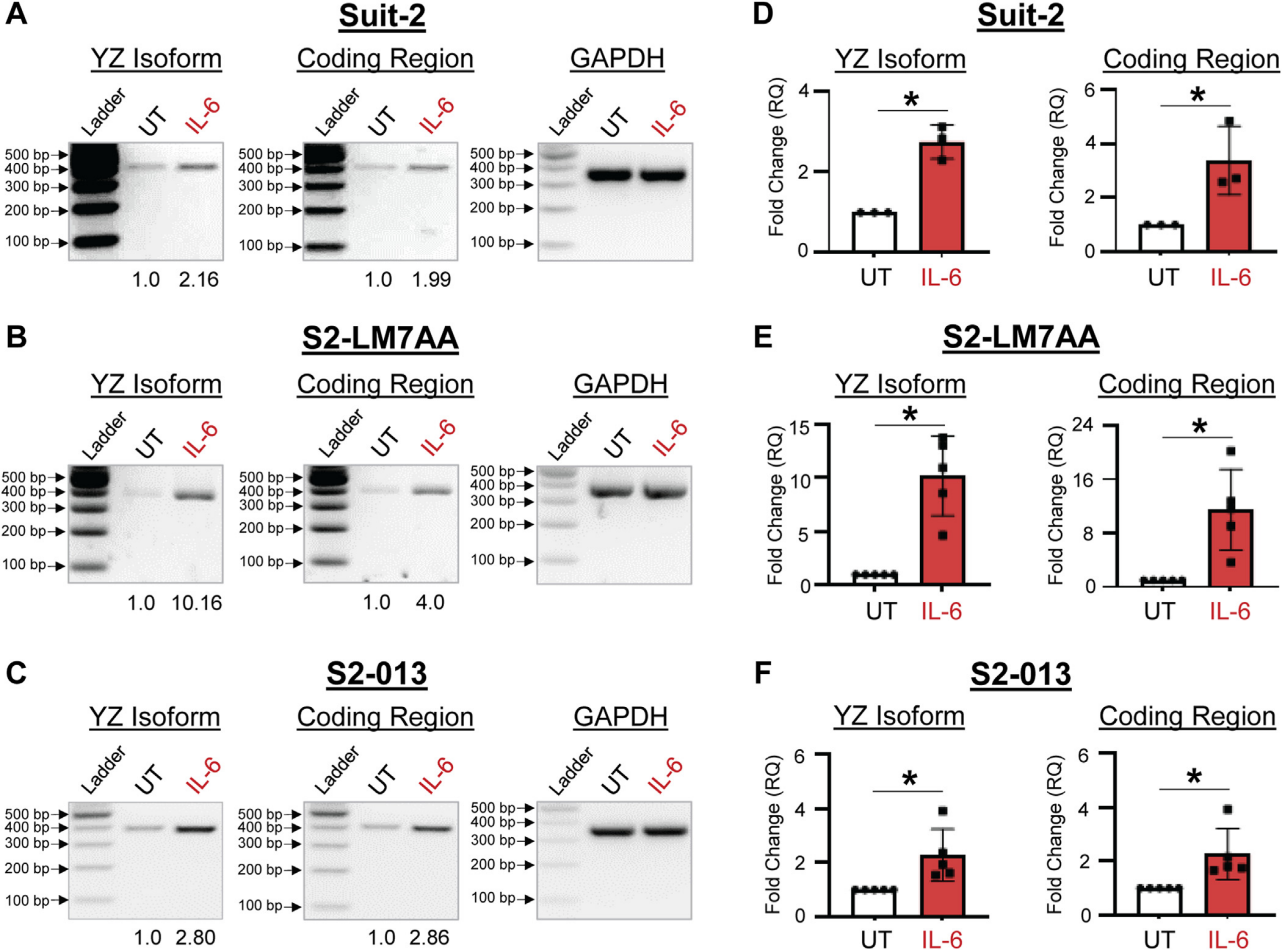
**IL-6 induces an upregulation in the YZ isoform of *ST6GAL1*.***A–C*, RNA was isolated from cells treated for 48 h with 25 ng/ml IL-6 or left untreated (UT). The YZ isoform and coding region of *ST6GAL1* were amplified by RT-PCR, and the PCR products resolved on agarose gels. Bands were quantified by densitometry. Densitometric units for the YZ and coding regions were normalized to GAPDH, and then values were reported as relative to the UT control cells. As shown, IL-6 treatment increased the expression of the YZ isoform and coding region in Suit-2 (*A*) S2-LM7AA (*B*), and S2-013 (*C*) cells. Predicted size of PCR products: YZ isoform = 363 bp; coding region = 372 bp; GAPDH = 371 bp. *D–F*, the YZ isoform and coding region were amplified by RT-qPCR using SYBR green. IL-6 treatment significantly increased the expression of the YZ isoform and coding region in Suit-2 (*D*, n = 3 biological replicates), S2-LM7AA (*E*, n = 5 biological replicates), and S2-013 (*F*, n = 5 biological replicates) cells. Graphs depict mean ± S.D. Data were analyzed using a two-tailed Student’s *t* test. ∗*p* < 0.05.

We next examined the effects of IL-1β and IL-6 on expression of the YZ isoform. Pilot studies were initially conducted to determine the optimal time interval for cytokine treatment (Fig. S1). These studies revealed that maximal increases in *ST6GAL1* mRNA were achieved following a 24-h incubation with IL-1β, and 48-h incubation with IL-6. These time points were used for subsequent experiments. Cells were treated with 10 ng/ml IL-1β, and then RT-PCR was performed, followed by gel electrophoresis. As shown in Fig. 5, *A*–*C*, IL-1β stimulated an increase in the YZ isoform and coding region for all three cell lines, as indicated by densitometric analyses. These results were validated by RT-qPCR, which confirmed elevated expression of the YZ isoform and coding region in IL-1β-treated cells (Fig. 5, *D–F*). We then examined the effects of IL-6. For all three PDAC lines, cells treated with 25 ng/ml IL-6 had enhanced levels of the YZ isoform and coding region as indicated by RT-PCR/gel electrophoresis (Fig. 6, *A–C*) and RT-qPCR (Fig. 6, *D–F*). The combined results in Figures 5 and 6 revealed that IL-1β and IL-6 induced a significant increase in ST6GAL1 expression in PDAC cells. However, we were also interested in whether ST6GAL1 expression was regulated by cytokines in nonmalignant pancreatic epithelial cells. We therefore tested the effects of IL-1β and IL-6 using the hTERT-HPNE (TERT-immortalized human pancreatic nestin-expressing) pancreatic epithelial cell line. Consistent with results from the PDAC lines, expression of the YZ isoform was increased in hTERT-HPNE cells by IL-1β and IL-6 (Fig. S2). These data point to a potential mechanism by which ST6GAL1 expression may be increased in nonmalignant acinar cells during pancreatitis.

As noted previously, no basal expression of the H isoform was observed in PDAC cells (Fig. 4, *B–D*). Nonetheless, we evaluated the effects of cytokines on this isoform, in light of prior evidence indicating that IL-6 stimulates expression of the H isoform in hepatocytes (44). However, there was no detectable expression of the H isoform following IL-1β or IL-6 treatment of the three PDAC cell lines (Fig. S3). Likewise, no expression of the X isoform was observed (Fig. S4), which aligns with the well-known restriction of this isoform to B lymphocytes (45). Thus, the YZ isoform was the major *ST6GAL1* transcript upregulated by IL-1β and IL-6 in PDAC cells.

### IL-1β and IL-6 stimulate the expression of ST6GAL1 through the NFκB and STAT3 transcription factors, respectively

**Figure 7:**
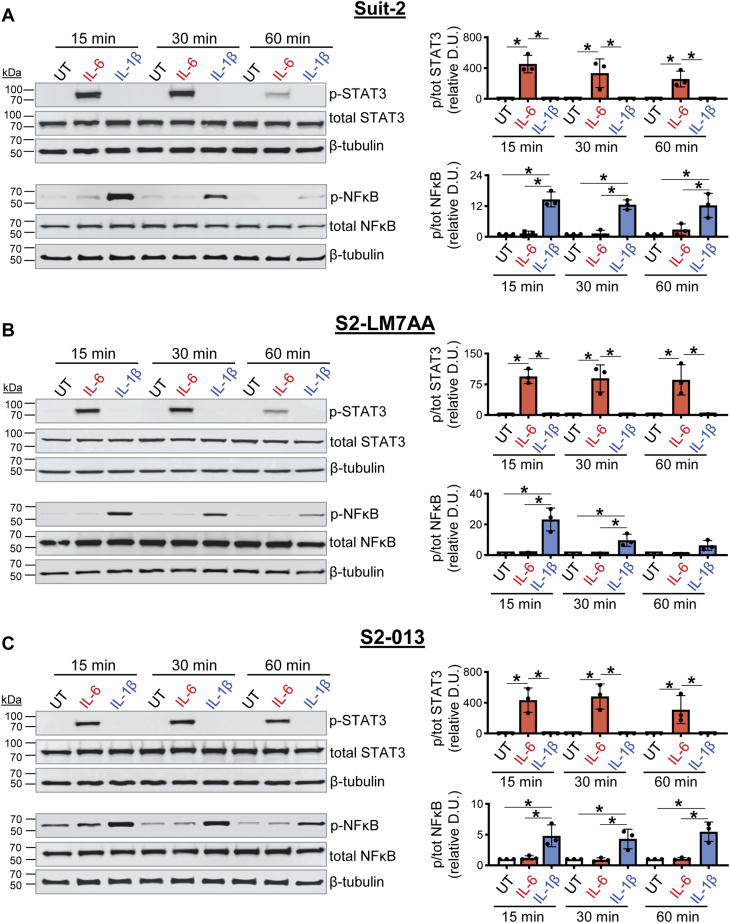
**Cytokine-induced activation of STAT3 and NFκB (p65).***A–C*, Suit-2, S2-LM7AA, and S2-013 cells were treated with IL-1β or IL-6 for 15, 30, or 60 min or left untreated (UT). Cell lysates were immunoblotted for total and phosphorylated (p-) STAT3 (pY705), as well as total and phosphorylated (p-) NF-κB p65 (pS536). IL-1β induced the activation of NFκB, while IL-6 induced the activation of STAT3 in Suit-2 (*A*), S2-LM7AA (*B*), and S2-013 (*C*) cells. Densitometry was conducted to obtain ratios for p-STAT3/total STAT3 and p-NFκB/total NFκB. Values were normalized to β-tubulin and depicted as relative to UT cells. Graphs depict mean ± S.D. (n = 3 biological replicates). The data were analyzed by one-way ANOVA followed by Tukey’s test. ∗*p* < 0.05.

We investigated the downstream signaling pathways through which IL-1β and IL-6 stimulated ST6GAL1 expression. IL-1β is known to signal through NFκB, whereas IL-6 mediates its effects *via* STAT3 (46, 47). To verify that these signaling pathways were operative in PDAC cells, Suit-2, S2-LM7AA, and S2-013 cells were treated with IL-1β or IL-6 and monitored for activation (phosphorylation) of NFκB and STAT3 by immunoblotting. IL-1β treatment activated NFκB, while IL-6 activated STAT3 (Fig. 7, *A–C*).

**Figure 8:**
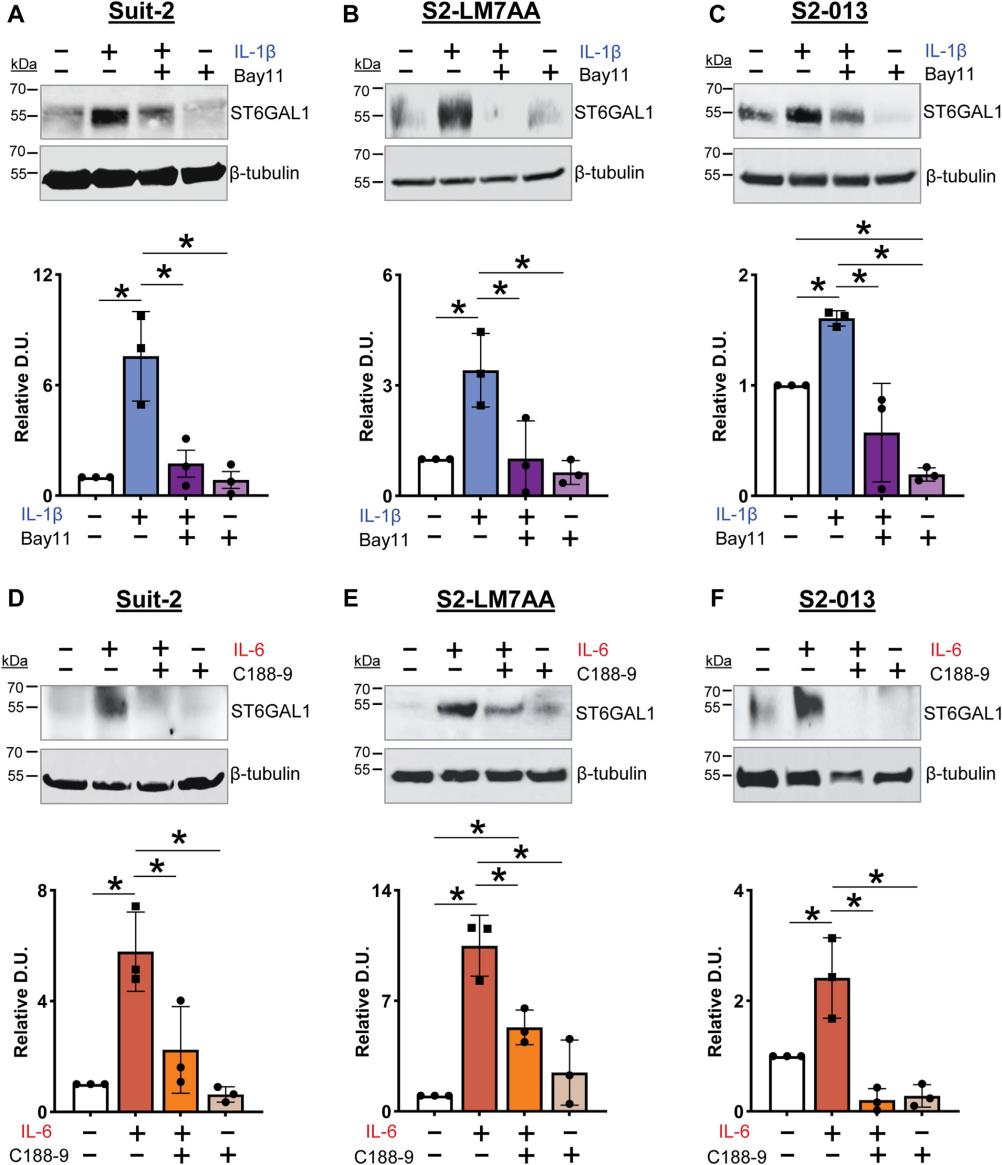
**Inhibitors of NFκB and STAT3 block the upregulation of ST6GAL1 stimulated by IL-1β and IL-6, respectively.***A–C*, pretreatment with the NFκB inhibitor, Bay11, blocks the IL-1β-induced upregulation of ST6GAL1 in Suit-2 (*A*), S2-LM7AA (*B*), and S2-013 (*C*) cells. *D–F*, pretreatment with the STAT3 inhibitor, C188-9, inhibits the IL-6 induced upregulation of ST6GAL1 in Suit-2 (*D*), S2-LM7AA (*E*), and S2-013 (*F*) cells. Densitometric values (D.U.) were normalized to β-tubulin and depicted as relative to untreated (UT) cells. Graphs depict mean ± S.D. (n = 3 biological replicates). The data were analyzed by one-way ANOVA followed by Tukey’s test. ∗*p* < 0.05.

To test whether these pathways were responsible for mediating cytokine-induced ST6GAL1 expression, we utilized a NFκB inhibitor, Bay11-7082 (Bay11), and a STAT3 inhibitor, C188-9. Bay11 impedes the nuclear translocation of NFκB by blocking the degradation of IKBa (48), while C188-9 inhibits STAT3 phosphorylation, which prevents its nuclear translocation (49). Cells treated with IL-1β displayed an increase in ST6GAL1 protein expression, which was attenuated by the NFκB inhibitor, Bay11 (Fig. 8, *A–C*). Similarly, the increase in ST6GAL1 protein expression induced by IL-6 was inhibited by the STAT3 inhibitor, C188-9 (Fig. 8, *D–F*).

### IL-1β and IL-6 stimulate transcription from the ST6GAL1 P3 promoter

**Figure 9:**
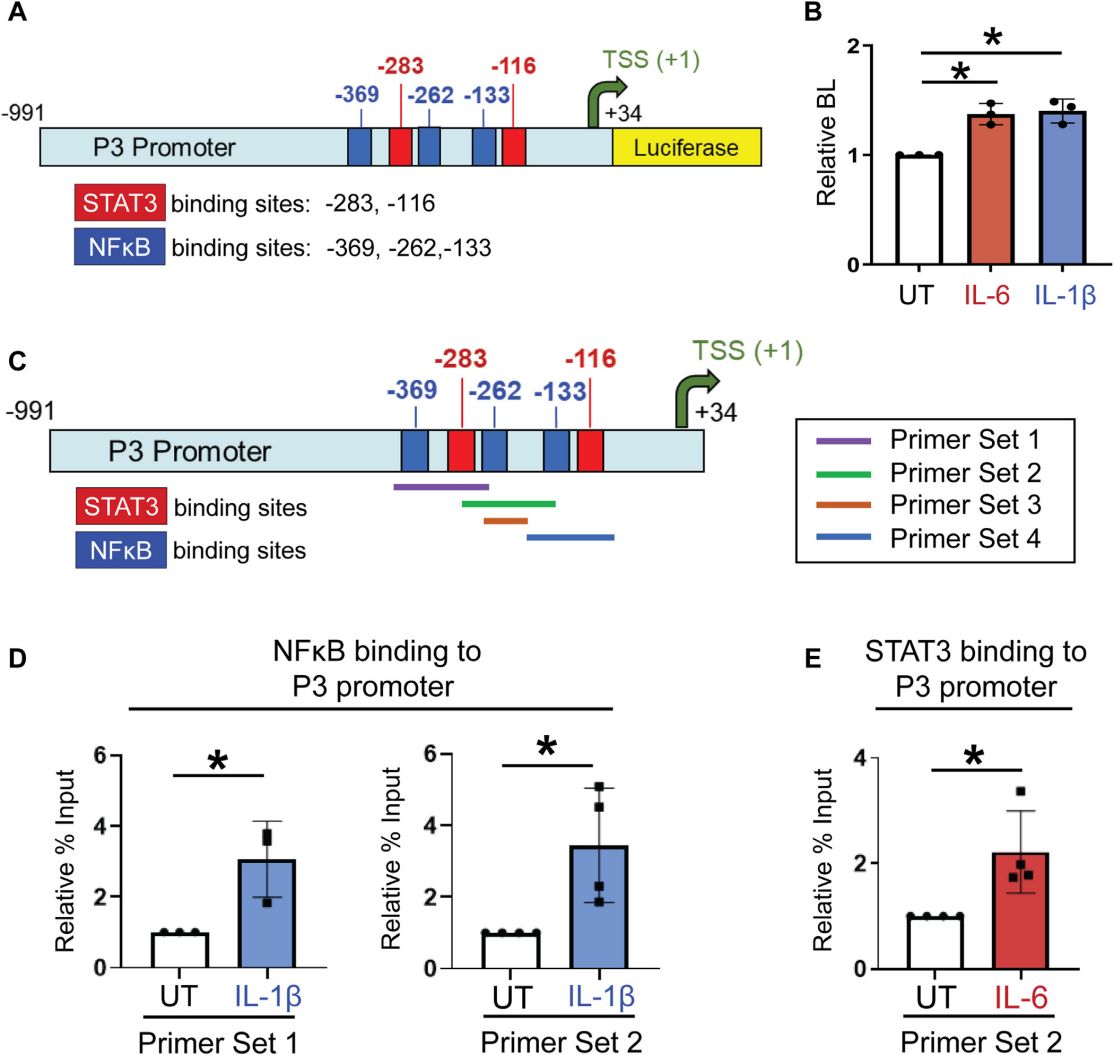
**The IL-1β/NFκB and IL-6/STAT3 signaling nodes activate transcription from the *ST6GAL1* P3 promoter.***A*, schematic diagram of the P3 promoter reporter construct. The diagram depicts the positions of putative binding sites for NFκB and STAT3, as indicated by the Gene Transcription Regulation Database. *B*, S2-LM7AA cells transfected with the P3 promoter construct were treated with IL-1β or IL-6 or left untreated (UT). Transcriptional activity was quantified by measuring bioluminescence (BL). Cells treated with IL-1β or IL-6 had significantly greater luciferase activity as compared with UT cells, consistent with cytokine-induced transcription from the P3 promoter. Graphs depict mean ± S.D (n = 3 biological replicates). The data were analyzed using a one-way ANOVA followed by Tukey’s test. ∗*p* < 0.05. *C*, schematic diagram of primer sets used in CUT&RUN assays. Four distinct sets of primers were designed to span the various NFκB and STAT3 binding sites on the *ST6GAL1* P3 promoter. *D*, S2-LM7AA cells were treated with IL-1β or left UT, and then CUT & RUN assays were performed to measure binding of NFκB to the P3 promoter. IL-1β induced the binding of NFκB to regions of the P3 promoter detected by Primer Sets 1 and 2. *E*, S2-LM7AA cells were treated with IL-6 or left UT, and then CUT & RUN assays were performed to measure binding of STAT3 to the P3 promoter. IL-6 induced the binding of STAT3 to a region of the P3 promoter detected by Primer Set 2. Graphs in panels *D* and *E* depict mean ± S.D. (n = 3 or n = 4 biological replicates). The data were analyzed using a two-tailed Student’s *t* test. ∗*p* < 0.05.

Luciferase reporter assays were employed to verify that IL-1β and IL-6 induced transcription of the P3 promoter-driven YZ isoform. We used a reporter construct that contains the P3 promoter along with a small region of the Y exon downstream of the TSS (Fig. 9*A*). The P3 promoter has multiple binding sites for NFκB and STAT3, as indicated by the Gene Transcription Regulation Database (Fig. 9*A*). S2-LM7AA cells were transfected with the reporter construct and luciferase activity was quantified by measuring bioluminescence. Cells treated with IL-1β or IL-6 displayed significantly greater luciferase activity as compared with cells not treated with cytokines (Fig. 9*B*), suggesting that IL-1β and IL-6 promote ST6GAL1 expression through a transcriptional mechanism.

### IL-1β and IL-6 induce the binding of NFκB and STAT3, respectively, to the P3 promoter

We hypothesized that NFκB and STAT3 bind directly to the P3 promoter to induce transcription of the *ST6GAL1* YZ isoform. To address this hypothesis, S2-LM7AA cells were treated with IL-1β or IL-6, and CUT & RUN assays were performed. RT-qPCR was conducted on the extracted DNA bound to NFκB or STAT3. Four distinct sets of primers, spanning the NFκB and STAT3 binding sites on the P3 promoter, were used for RT-qPCR (Fig. 9*C*). In cells treated with IL-1β, CUT&RUN assays for NFκB binding revealed a significant increase in the products amplified by Primer Set one and Primer Set 2 (Fig. 9*D*). In cells treated with IL-6, assays for STAT3 binding showed a significant increase in the product amplified by Primer Set 2 (Fig. 9*E*). Data for all of the Primer Sets are shown in Fig. S5. In the aggregate, these results demonstrate that ST6GAL1 is transcriptionally upregulated by IL-1β acting through NFκB and IL-6 acting through STAT3.

## Discussion

The pro-inflammatory cytokines IL-1β and IL-6 are abundant within the PDAC TME and are associated with a poor prognosis (37, 50, 51). Interventions that block signaling by IL-1β and IL-6 suppress tumor growth in various mouse models of PDAC (38, 52, 53). For example, function-blocking antibodies against the IL-6 receptor, IL-6R (Tocilizumab), inhibit PDAC growth and metastatic progression, leading to enhanced overall survival (52, 53). One of the key mechanisms by which these cytokines promote pathogenesis is through creating an immunosuppressive microenvironment (38, 39, 54, 55, 56, 57). Additionally, tumor cells exposed to IL-1β and IL-6 acquire an invasive and chemoresistant phenotype (52, 58, 59, 60, 61, 62, 63, 64).

IL-1β and IL-6 have been previously linked to the regulation of ST6GAL1 expression. Bassaganas *et al.* (65) reported that IL-1β increased the expression of *ST6GAL1* mRNA in pancreatic cancer cell lines. However, this study did not identify the specific *ST6GAL1* mRNA isoform nor was ST6GAL1 upregulation confirmed at the protein level. Furthermore, the signaling mechanisms downstream of IL-1β were not explored. In the case of IL-6, it was shown that ST6GAL1 expression was increased in the liver by IL-6 during the acute phase response (44). Specifically, IL-6 was found to activate the HNF1A transcription factor which, in turn, bound to the P1 promoter to stimulate transcription of the *ST6GAL1* H isoform in hepatocytes. The H isoform has historically been considered to be liver-specific, in part, because of the selective expression of HNF1A in the liver. However, recent studies suggest that HNF1A is also expressed in the pancreas (66). Based on this finding, we speculated there might be some expression of the H isoform in pancreatic cancer cells. However, the YZ isoform was the only isoform detected in the PDAC cell lines used in the current study.

To interrogate the mechanisms by which IL-1β and IL-6 stimulate the expression of ST6GAL1, we examined the activity of canonical transcription factors downstream of IL-1β and IL-6, NFκB and STAT3, respectively. Using a combination of promoter reporter and CUT&RUN assays, we determined that transcription of the *ST6GAL1* YZ isoform is driven by IL-1β signaling through NFκB and IL-6 signaling *via* STAT3. Importantly, inhibitors of NFκB and STAT3 ablated the cytokine-induced expression of ST6GAL1 protein. The IL-1β/NFκB and IL-6/STAT3 signaling networks have well-established tumor-promoting functions. High expression of IL-1β in the TME promotes the sustained activation of NFκB (51), which contributes to tumor initiation. NFκB activation in neoplastic cells increases the expression of many protumorigenic genes including cell cycle regulators and antiapoptotic proteins (51). Additionally, activated NFκB is one of the main molecular drivers of cancer stem cell characteristics (67). Similar to NFκB, STAT3 is highly activated in PDAC cells, and expression of p-STAT3 is a negative prognostic marker (68, 69). In the Kras^G12D^-expressing PDAC mouse model, genetic deletion of STAT3 inhibited both ADM and the development of premalignant pancreatic intraepithelial neoplasms (70, 71). STAT3 deficiency also suppressed the progression of pancreatic intraepithelial neoplasms to PDAC (70, 71).

The molecular pathways that regulate ST6GAL1 expression remain poorly understood. One important prior finding is that ST6GAL1 expression is upregulated by the ras oncogene (72, 73, 74). Dalziel *et al.* reported that oncogenic ras signals through a ralGEF-dependent axis to stimulate transcription of the *ST6GAL1* YZ isoform (73). In other studies, Lu *et al.* (15) determined that TGFβ-driven epithelial to mesenchymal transition promoted ST6GAL1 expression *via* the Sp1 transcription factor. In tandem with transcriptional activation, ST6GAL1 is upregulated in cancer cells through gene amplification. Increases in *ST6GAL1* gene copy number are observed in many different types of malignancies, including 30 to 40% of lung cancer patients (75). Epigenetic regulation of ST6GAL1 expression has also been described, although more work is needed in this area. The P3 promoter for the *ST6GAL1* YZ isoform has a prominent CpG island that is reportedly hypermethylated in certain types of cancer, including glioblastoma and bladder cancer (76, 77). It was suggested that promoter hypermethylation correlated with reduced ST6GAL1 expression (76, 77). However, more recent studies examining ST6GAL1 protein expression have indicated that ST6GAL1 is upregulated in glioblastoma and bladder cancer (78, 79), consistent with the overexpression of ST6GAL1 in most other cancer types. As a final regulatory mechanism, the 3′UTR of *ST6GAL1*, which is conserved among mRNA isoforms, has putative binding sites for multiple miRNAs (80, 81, 82). Interestingly, Jame-Chenarboo *et al.* reported that most of the miRNAs that bind to the *ST6GAL1* 3′-UTR stimulated an increase, rather than decrease, in ST6GAL1 expression (82). Furthermore, the miRNA-dependent upregulation of ST6GAL1 was accompanied by increased cell surface α2-6 sialylation, confirming enhanced ST6GAL1 activity (82).

The studies described above offer important insights into tumor cell-intrinsic mechanisms that promote ST6GAL1 overexpression; however, a significant gap remains in our knowledge of extrinsic factors in the TME that impact ST6GAL1 expression. The current finding that ST6GAL1 expression is induced by IL-1β and IL-6 suggests that the inflammatory milieu associated with PDAC may act in concert with tumor-intrinsic mechanisms to promote high levels of ST6GAL1 expression. Our results also point to a potential mechanism by which ST6GAL1 expression may be increased in nonmalignant acinar cells by inflammatory cytokines produced during pancreatitis. We previously reported selective expression of ST6GAL1 in ADM-like cells during pancreatitis and established ST6GAL1 as a functional driver of ADM (11). Given the importance of ADM to pancreatic regeneration following inflammatory damage, it is tempting to speculate that ST6GAL1 may function in tissue repair. In support of this concept, Punch *et al.* demonstrated that mice with deletion of ST6GAL1 succumbed to whole body radiation because they were unable to regenerate their intestinal tract following radiation-induced damage (83). More studies will be needed to elucidate the biological consequences of ST6GAL1 upregulation in nonmalignant pancreatic epithelial cells exposed to inflammation and other assaults.

In sum, the present investigation provides a conceptual advance by highlighting a new regulatory mechanism that contributes to the increased expression of ST6GAL1 in tumor cells exposed to an inflammatory milieu. Given the well-known role of ST6GAL1 in promoting cancer initiation and progression, results herein may point to novel avenues for therapeutic intervention by illuminating strategies for preventing or suppressing the overexpression of ST6GAL1 in malignant cells.

## Experimental procedures

### Immunohistochemistry on pancreatic tissues

Formalin-fixed, paraffin-embedded pancreatic tissues from patients with chronic pancreatitis or PDAC were obtained from the UAB Tissue Biorepository. Nonaffected, adjacent tissues associated with the pathological specimens were used as the “normal” controls. Tissue sections were subjected to antigen retrieval using Antigen Unmasking Solution, Citric Acid Based (Vector Laboratories, H-3300), blocked with 2.5% horse serum for 1 h, and incubated overnight at 4 °C with primary antibody against IL-6 (1:500, Abcam, ab6672) or IL-1β (1:500, Thermo Fisher, P420B). Sections were subsequently incubated with ImmPRESS-HRP anti-rabbit IgG (Vector Laboratories, 30026) for 1 h and developed using ImmPACTDAB (Vector Laboratories, SK-4105). Images were captured with a Nikon 80i Eclipse microscope and processed with Nikon NIS-Elements imaging software.

### Immunofluorescent staining on pancreatic tissues

Formalin-fixed, paraffin-embedded pancreatic tissue sections were subjected to antigen retrieval as described above and then blocked with 2.5% horse serum for 1 h. Specimens were incubated overnight at 4 °C with antibodies against IL-6 (1:50, Abcam, ab6672), IL-1β (1:200, Thermo Fisher, P420B), and/or ST6GAL1 (1:75, R&D Systems, AF5924). Sections were incubated with the appropriate secondary antibodies, specifically, anti-rabbit IgG-Alexa Fluor 488 (1:1000, Thermo Fisher, A32790) or anti-goat IgG-Alexa Fluor 546 (1:1000, Thermo Fisher, A-11056) for 1.5 h at 37 °C. This was followed by incubation in Hoechst solution (Thermo Fisher, H3570) for 5 min. Slides were mounted using VECTASHIELD Vibrance Antifade Mounting Medium (Vector Laboratories, H-1700), and images were captured with a Nikon 80i Eclipse microscope and processed with Nikon NIS-Elements imaging software.

### Cell culture

Suit-2 and S2-013 cells were donated by Dr Michael A. Hollingsworth at the University of Nebraska. The S2-LM7AA subclone was developed by Drs Lacey McNally and Donald Buchsbaum at the University of Alabama at Birmingham. hTERT-HPNE cells were purchased from the American Type Culture Collection (CRL-4023). Suit-2 parental and metastatic subclones were maintained in RPMI medium containing 10% fetal bovine serum (FBS) and 1% antibiotic/antimycotic supplements (Gibco). hTERT-HPNE cells were maintained in 75% Dulbecco's modified Eagle's medium without glucose and 25% Medium M3 Base (Incell Corporation, M300F- 500) supplemented with 2 mM L-glutamine, 1.5 g/l sodium bicarbonate, 5.5 mM D-glucose, 750 ng/ml puromycin, 10 ng/ml human recombinant EGF (Thermo Fisher, PHG0311), and 5% FBS. Recombinant IL-1β (Thermo Fisher, PHC0811) and IL-6 (Thermo Fisher, 8954SR025CF) were reconstituted in saline containing 0.1% bovine serum albumin (BSA). For cytokine treatments, cells were preincubated in serum-free media for 2 h and then treated for the indicated times with a final concentration of 10 ng/ml IL-1β or 25 ng/ml IL-6, added to 2% FBS-containing media. For experiments using the STAT3 and NFκB inhibitors, cells were pretreated for 2 h with either 12 μM C188-9 (Selleckchem, S8605) or 7 μM Bay11-7082 (Selleckchem, S2913) in serum-deficient media. IL-1β or IL-6 was then added to the inhibitor-containing media, or control media, for the indicated times.

### Immunoblotting

Cells were lysed using radioimmunoprecipitation assay buffer (RIPA) (Pierce, 89901) with protease and phosphatase inhibitors (Pierce, 78440). Protein concentration was measured using BCA (Pierce, 23225). Samples were resolved by SDS-PAGE and transferred to polyvinylidene difluoride membranes. Following transfer, membranes were blocked with 5% nonfat dry milk in Tris-buffered saline containing 0.1% Tween 20 (TBST). Immunoblots were probed with antibodies against ST6GAL1 (1:500, R&D Systems, AF5924), p-STAT3 (pY705, 1:1000, Cell Signaling Technologies, 9145), p-NFκB p65 (pS536, 1:1000, Cell Signaling Technologies, 3033), STAT3 (1:1000, Cell Signaling Technologies, 9139), and NFκB p65 (1:1000, Cell Signaling Technologies, 8242). Following primary antibody incubation, membranes were washed in TBST and incubated with HRP-conjugated secondary antibodies, either anti-rabbit IgG (1:2500, Cell Signaling Technologies, 7074), anti-mouse IgG (1:2000, Cell Signaling Technologies, 7076), or anti-goat IgG (1:2000, R&D Systems, HAF109). Equal protein loading was confirmed by blotting with antibody against β-tubulin (1:2500, Abcam, ab21058). Blots were developed with Clarity Western ECL Substrate (Bio-Rad, 1705061) or SuperSignal West Femto (Pierce 34096). Densitometric values were calculated using ImageJ and normalized to β-tubulin. Densitometric analyses were conducted on three independent blots (*i.e.*, from three independently generated cell lysates).

### SNA staining and flow cytometry

Cells treated with or without IL-1β or IL-6 were detached with accutase (BioLegend, 423201) and blocked on ice with 1% BSA in PBS. Cells were washed with 0.01% BSA in PBS and then incubated for 30 min on ice with SNA-Cy5 (Vector Laboratories, CL-1305-1). SNA-Cy5 was used at a dilution of 1:100 with the following exceptions: S2-LM7AA cells treated with or without IL-6 were incubated with 1:400 SNA-Cy5, and S2-013 cells treated with or without IL-1β were incubated with 1:50 SNA-Cy5. Data were analyzed using FlowJo, version 8, software (BD Biosciences).

### RT-PCR of ST6GAL1 mRNA isoforms

RNA was extracted using the Qiagen RNeasy kit (Qiagen, 74106) in accordance with the manufacturer’s protocol. RNA concentration was measured, and cDNA was synthesized using M-MLV reverse transcriptase (Promega, M1701). RT-PCR was performed with primers specific for the various ST6GAL1 isoforms. PCR products were visualized on 2% agarose gels with GelRed Nucleic Acid Stain (Thermo Fisher, SCT123). PCR products were quantified by densitometry using ImageJ, and values were normalized to GAPDH. The primers (Integrated DNA Technologies) for the various isoforms contained the following sequences:

**Table:** 

YZ isoform:	Forward: AGTCCAGGGAGAAGTGGTGA
	Reverse: CCACACACAGATGACTGCAA
H isoform:	Forward: TGTCTCTTATTTTTTGCCTTTG
	Reverse: CCACACACAGATGACTGCAA
X isoform:	Forward: CTTCTCCCATACCTTGCTCTACA
	Reverse: GAAGATGTGTTCAGGGAAGTCAC
Coding:	Forward: TATCGTAAGCTGCACCCCAATC
	Reverse: TTAGCAGTGAATGGTCCGGAAG
GAPDH:	Forward: TGGTATCGTGGAAGGACTCA
	Reverse: AGTGGGTGTCGCTGTTGAAG

### RT-qPCR of ST6GAL1 mRNA isoforms

For RT-qPCR analysis of the ST6GAL1 YZ isoform and coding region, SYBR Green Mastermix (Thermo Fisher, 4309155) was used. At least three independent RT-qPCR experiments were conducted, with each experiment performed with three technical replicates. Primer sequences for the YZ isoform and coding region are listed above. Values were normalized to β-actin mRNA using the following primers:

**Table:** 

β-actin:	Forward: GGATGCAGAAGGAGATCA,
	Reverse: CTAGAAGCATTTGCGGTG

### Luciferase reporter assays

To monitor ST6GAL1 P3 promoter activity, we used a construct with the P3 promoter sequence conjugated to the *Renilla reniformis* luciferase reporter gene (Active Motif, LightSwitch luciferase assay, S707077). Cells were transfected with this construct using the FuGENE HD transfection reagent (FuGENE, HD-1000) according to the manufacturer’s protocol. To confirm transfection efficiency and specificity of the reactions, cells were also transfected with a GAPDH promoter reporter construct (positive control, Active Motif, LightSwitch luciferase assay, 32004) and a random genomic fragments promoter construct (negative control, Active Motif, LightSwitch luciferase assay, 32007). Following transfection with the various constructs, cells were treated with IL-1β or IL-6 for 6 h. Luminescence was measured using a microplate reader, and values were normalized to the GAPDH construct. Data are reported as relative luminescence units as compared with cells not treated with cytokines.

### CUT & RUN assays

S2-LM7AA cells were treated with or without IL-1β or IL-6 for 6 h, and then protein–DNA interactions were analyzed using the CUT&RUN assay kit (Cell Signaling Technologies, 86652). Assays were performed with ∼100,000 cells per reaction. CUT&RUN assays were conducted using antibodies against STAT3 (Cell Signaling Technologies, 9139T) or NFκB p65 (Cell Signaling Technologies, 8242T). An antibody against trimethylated histone H3 (Lys4) (H3K4me3, Cell Signaling Technologies, 9751) was used as the positive control, and an IgG isotype control antibody (Cell Signaling Technologies, 66362) was used as the negative control. The extracted DNA from CUT&RUN reactions was purified using DNA spin columns (Cell Signaling Technologies, 14209S). The products were quantified by qPCR using primers for various regions within the P3 promoter sequence. Values were normalized to % input. Results for cytokine-treated cells were reported as relative % input units as compared to untreated cells. The following primer sets were used:

**Table:** 

Primer Set 1:	Forward: CCAGTGCCTTCATCAAGAGT
	Reverse: TCGCCTCCGTATGCAAATAG
Primer Set 2:	Forward: CCAGTCTTCGCAACTCTATTT
	Reverse: CACTAGGGAGTCTCGAAAGA
Primer Set 3:	Forward: CGCAACTCTATTTGCATACGG
	Reverse: GACTGGAGAAGGAAGGAAGAA
Primer Set 4:	Forward: ACTGTGCGTCTTCTGTC
	Reverse: CCAGGTGACTCCAGAAA

## Data availability

All data described in this study are contained within the manuscript or supplementary figures.

## Supporting information

This article contains supporting information.

## Conflict of interest

The authors declare that they have no conflicts of interest with the contents of this article.
