# Supplementary material for: The pro-inflammatory cytokines IL-1β and IL-6 promote upregulation of the ST6GAL1 sialyltransferase in pancreatic cancer cells
Source: J Biol Chem. 2024 Sep 12;300(10):107752. doi: 10.1016/j.jbc.2024.107752 (PMC11470512; doi:10.1016/j.jbc.2024.107752)
Supplement: Supplemental Figures S1–S5 [file mmc1.pdf]

The pro-inflammatory cytokines IL-1β and IL-6 promote upregulation of the ST6GAL1 sialyltransferase in pancreatic cancer cells

Austin D. Silva, Jihye Hwang, Michael P. Marciel, Susan L. Bellis*

Department of Cell, Developmental and Integrative Biology, University of Alabama at Birmingham, Birmingham, AL

Running title: IL-1β and IL-6 promote the upregulation of ST6GAL1

*** To whom correspondence should be addressed:**

Susan L. Bellis, Ph.D.

Department of Cell, Developmental and Integrative Biology

University of Alabama at Birmingham

Birmingham, AL 35294

(205) 934-3441

bellis@uab.edu

Material Included:

Figure S1. Time course for cytokine-induced expression of the *ST6GAL1* YZ isoform.

Figure S2. IL-1β and IL-6 promote the expression of the *ST6GAL1 YZ* isoform in the non-malignant pancreatic cell line, hTERT-HPNE.

Figure S3. IL-1β and IL-6 have no effect on expression of the *ST6GAL1* H isoform.

Figure S4. IL-1β and IL-6 have no effect on expression of the *ST6GAL1* X isoform.

Figure S5. CUT & RUN assays for cytokine-induced binding of NFκB and STAT3 to the *ST6GAL1* P3 promoter

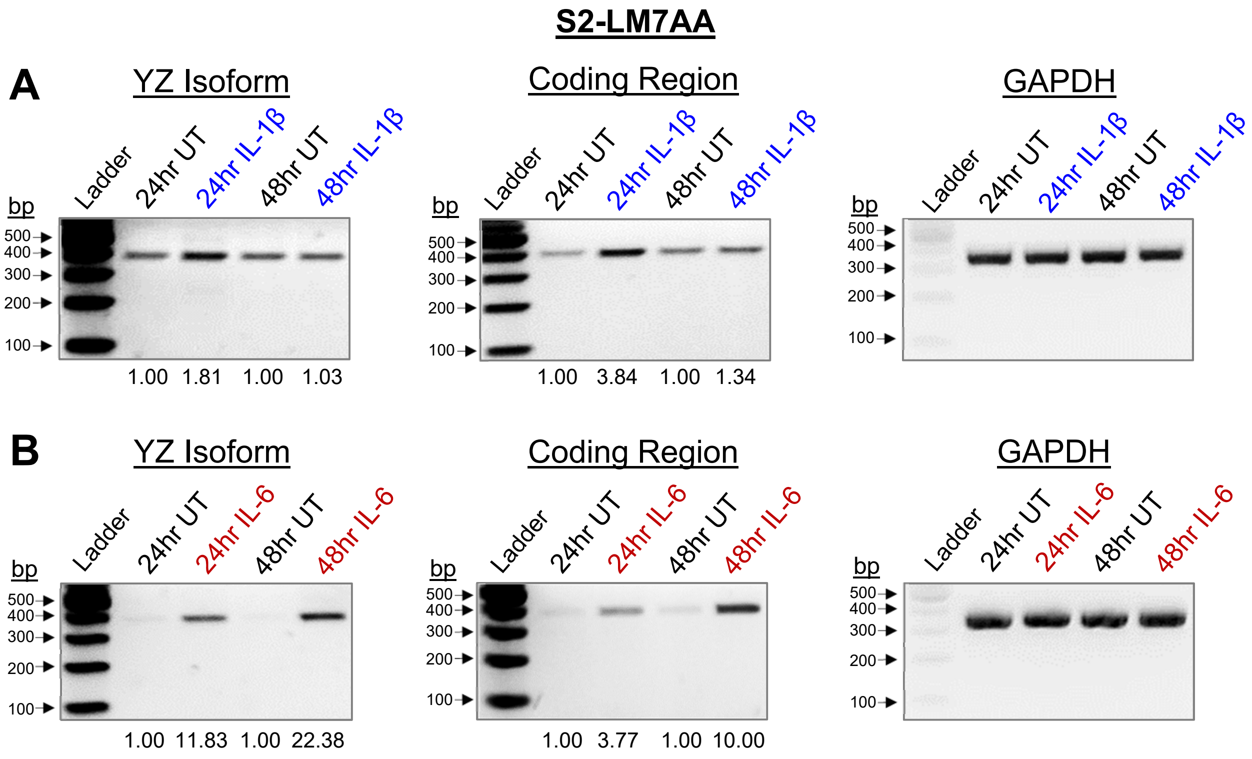

Figure S1. Time course for cytokine-induced expression of the *ST6GAL1* YZ isoform. *A-B*) S2-LM7AA cells were treated for 24 or 48 hr with IL-1β (*A*) or IL-6 (*B*). Levels of the YZ isoform and coding region of *ST6GAL1* were evaluated by RT-PCR, followed by gel electrophoresis. PCR products were quantified by densitometry, and normalized to GAPDH. Values are depicted as relative to untreated (UT) cells. Maximal increases in ST6GAL1 were noted at 24 hr after IL-1β treatment and 48 hr after IL-6 treatment. Expected band sizes: YZ isoform= 363 bp; coding region = 372 bp; GAPDH= 371 bp.

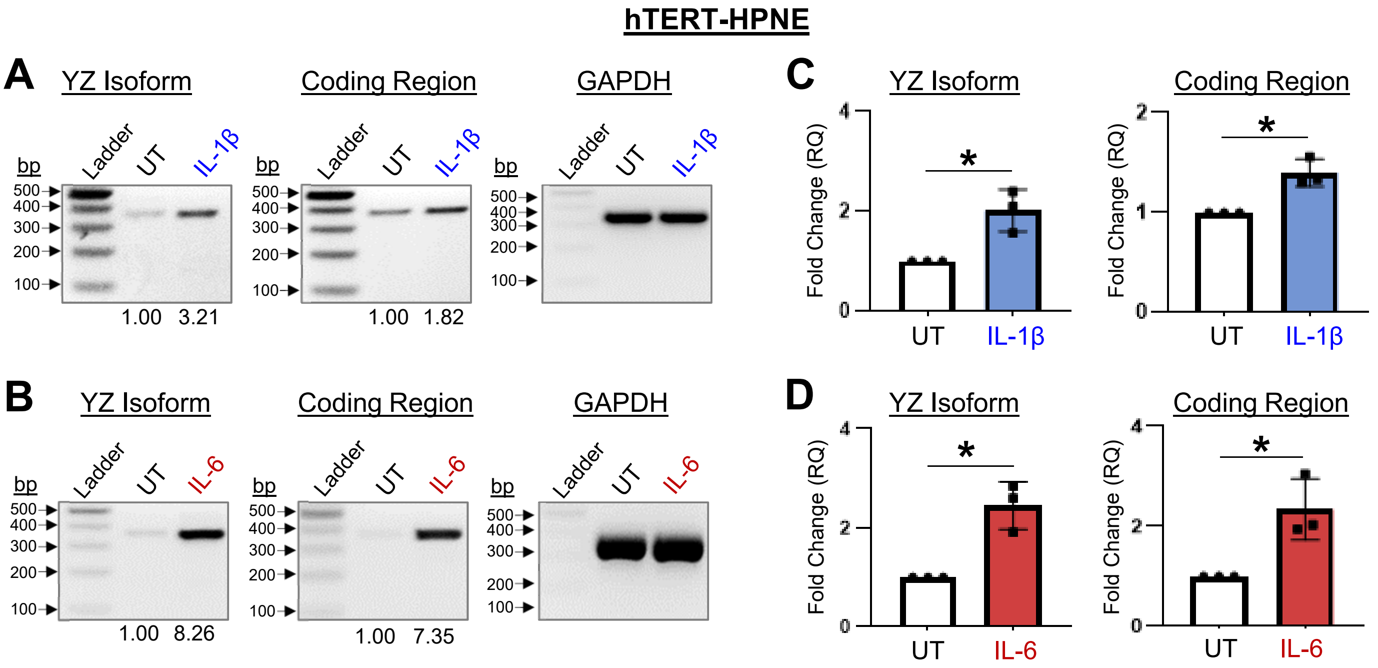

Figure S2. IL-1β and IL-6 promote the expression of the *ST6GAL1* YZ isoform in the non-malignant pancreatic acinar-like cell line, hTERT-HPNE. *A-B)* hTERT-HPNE cells were treated with IL-1β for 24 hr (*A*) or IL-6 for 48 hr (*B*). Levels of the YZ isoform and coding region of *ST6GAL1* were evaluated by RT-PCR and gel electrophoresis. Densitometric values for *ST6GAL1* were normalized to *GAPDH* and depicted as relative to untreated (UT) cells. Expected PCR products: YZ isoform= 363 bp; coding region = 372 bp; *GAPDH* = 371 bp. *C-D)* RT-qPCR was conducted on cytokine-treated cells using SYBR green. As shown, IL-1β (*C*) and IL-6 (*D*) significantly increased expression of the *ST6GAL1* YZ isoform and coding region. Graphs depict mean $\pm$ S.D. (n = 3 biological replicates). Data were analyzed using a two-tailed Student's t test. *$p < 0.05$.

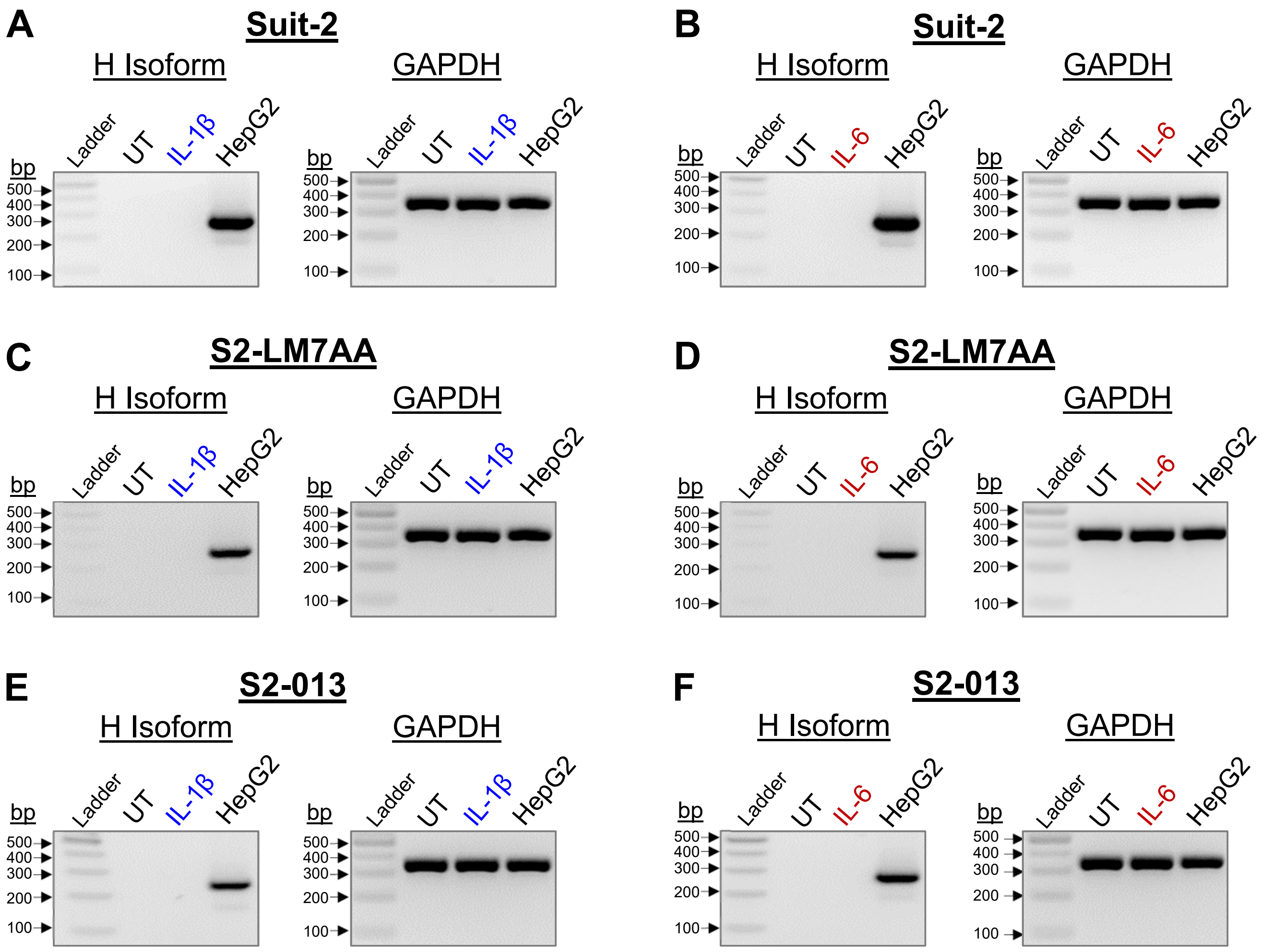

Figure S3. IL-1β and IL-6 have no effect on expression of the *ST6GAL1* H isoform. *A-F*) Cells were treated with IL-1β for 24 hr (*A,C,E*) or IL-6 for 48 hr (*B,D,F*) and evaluated for expression of the H isoform by RT-PCR/gel electrophoresis. The hepatocellular carcinoma cell line, HepG2, was used as a positive control for expression of the H isoform. No detectable H isoform was observed in either untreated (UT) or cytokine-treated cells. Expected band sizes: H isoform = 285 bp; *GAPDH* = 371 bp.

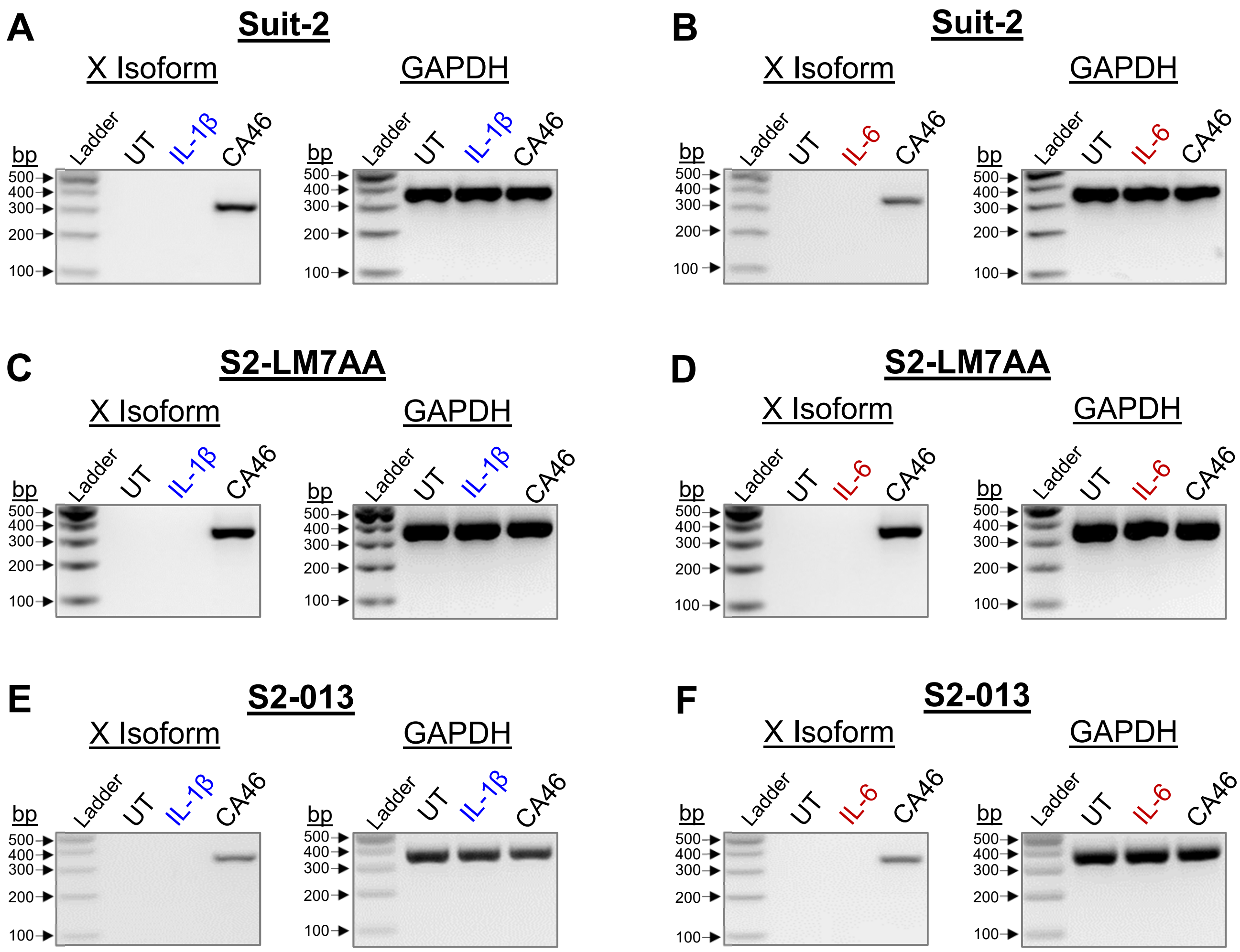

Figure S4. IL-1β and IL-6 have no effect on expression of the *ST6GAL1* X isoform.
A-F) Cells were treated with IL-1β for 24 hr (*A,C,E*) or IL-6 for 48 hr (*B,D,F*) and evaluated for expression of the X isoform by RT-PCR/gel electrophoresis. The Burkitt lymphoma cell line, CA46, was used as a positive control for expression of the X isoform. No detectable X isoform was observed in either untreated (UT) or cytokine-treated cells. Expected band sizes: X isoform = 334 bp; *GAPDH* = 371 bp.

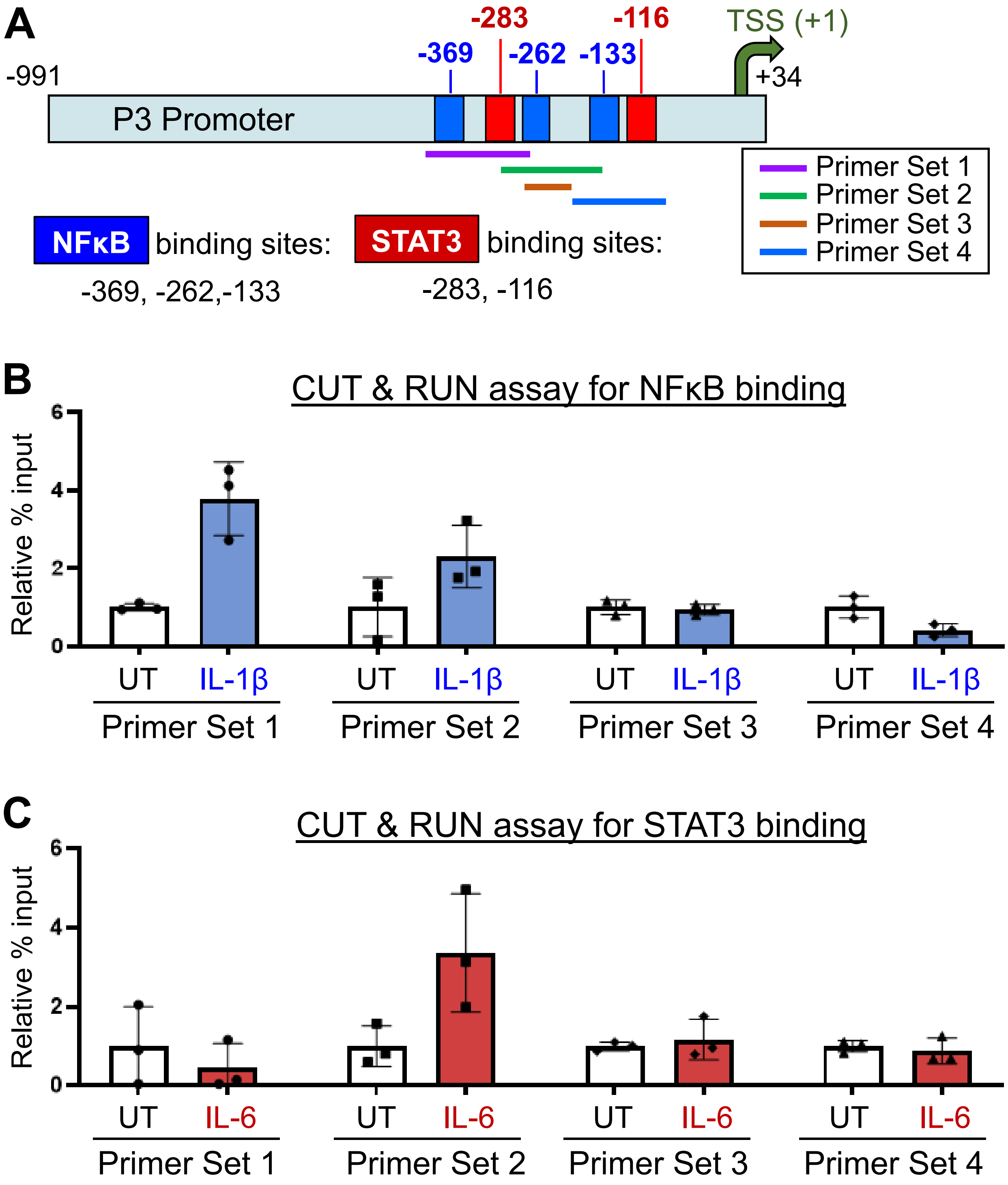

Figure S5. CUT & RUN assays for cytokine-induced binding of NFκB and STAT3 to the *ST6GAL1* P3 promoter. *A)* Four sets of primers were designed to span the predicted binding sites for NFκB and STAT3 on the *ST6GAL1* P3 promoter. *B)* CUT & RUN assays were performed on S2-LM7AA cells treated with IL-1β or left untreated (UT). IL-β treatment stimulated an increase in NFκB binding to sequences detected by Primer Sets 1 and 2, but not Primer Sets 3 and 4. Representative experiment is shown (n = 3 technical replicates). *C)* CUT & RUN assays were performed on S2-LM7AA cells treated with IL-6 or left untreated (UT). IL-6 treatment stimulated an increase in STAT3 binding to a sequence detected by Primer Set 2, but not Primer Sets 1,3 or 4. Representative experiment is shown (n = 3 technical replicates).
